# Structured, evidence-based debate in science: Introducing a new forum

**DOI:** 10.17179/excli2026-9618

**Published:** 2026-06-19

**Authors:** Agapios Sachinidis, Wiebke Albrecht, Jan G. Hengstler

**Affiliations:** 1 Center for Physiology, Faculty of Medicine and University Hospital Cologne, University of Cologne, Robert-Koch-Str. 39, 50931 Cologne, Germany; 2 Department of Toxicology, Leibniz Research Centre for Working Environment and Human Factors (IfADo), Ardeystr. 67, Dortmund, 44139, Germany; 3 Department of Toxicology, Faculty of Chemistry, TU Dortmund, 44227 Dortmund, Germany

## ⁯⁯⁯⁯

Scientific progress depends on the generation of new knowledge and on the continuous critical examination of the structures, practices, and assumptions that shape research itself. While mechanisms for evaluation and oversight in science are well established - ranging from peer review to funding decisions and institutional assessments - opportunities for structured, open, and evidence-based discussion of these mechanisms remain limited.

At the same time, there is a growing perception that raising critical questions about systemic aspects of scientific practice is becoming more difficult. Concerns related to publication processes, funding decisions, professional conduct, and institutional accountability are frequently discussed informally, yet rarely find their way into formal, citable discourse. This gap is not conducive to the long-term robustness and credibility of science.

With this in mind, we introduce a new forum dedicated to the structured discussion of science-associated topics. The aim of this forum is to enable rigorous, evidence-based analysis and constructive debate on issues that directly affect scientific work and working conditions.

We explicitly welcome contributions that address complex or sensitive issues, including those that may be perceived as controversial. The acceptability of a contribution will not depend on whether its conclusions align with prevailing views, but on the clarity of its reasoning, the quality of its evidence, and its adherence to scholarly standards. Divergent perspectives are essential to scientific progress - provided they are articulated in a transparent, rigorous, and respectful manner.

To ensure this standard, all factual claims, particularly those that may affect individuals, institutions, or organizations, must be supported by verifiable evidence. Contributors are expected to clearly distinguish between evidence-based statements, reasoned interpretation, and personal opinion. Unsupported allegations, speculation, or insinuations will not be considered. This framework is intended to safeguard both the integrity of the discussion and the fairness toward all parties involved.

The forum will follow a structured format designed to facilitate balanced and substantive exchange. Typically, a lead contribution presenting a clearly defined position will be followed by a response offering a critical or alternative perspective. Where appropriate, one or if required, two additional rounds of rebuttal may be included. The editorial team may provide a concluding synthesis to the discussion, without altering the substantive positions expressed. This structure is intended to enable readers to form an informed and independent judgment.

The role of the editorial team is limited to ensuring that contributions meet formal and scholarly standards, including clarity, evidentiary support, and adherence to the principles outlined above. Responsibility for the content, arguments, and conclusions remains with the authors. Substantive positions expressed in contributions will not be altered by the editors.

Topics of interest include, but are not limited to

- integrity and transparency in publication practices,
- fairness and accountability in funding decisions,
- professional conduct and the protection of early-career researchers,
- due process in allegations of misconduct, academic freedom and pressures toward intellectual conformity,
- potential external influences on research agendas.

Within this broader context, attention may also be given to situations in which scientists perceive procedural imbalances. This may include, for example, the handling of allegations of misconduct - especially in cases where allegations are raised without a transparent evidentiary basis, or where procedural frameworks limit the opportunity for timely and effective response by affected individuals.

By establishing this forum, we aim to strengthen scientific discourse by enabling transparent, evidence-based debate on systemic challenges in science. In doing so, we hope to contribute to the self-correcting capacity and credibility of scientific practice. We invite the scientific community to engage with this initiative in the spirit of rigor and intellectual openness that defines science at its best and underpins its continued advancement.

## Declaration

### Artificial Intelligence (AI) - assisted technology

No AI tools were used in the preparation of this Editorial.

### Conflict of interest

The authors are editors of EXCLI Journal. They declare no competing financial interests.

